# Pleural Effusion Caused by *Mycolicibacterium mageritense* in an Immunocompetent Host: A Case Report

**DOI:** 10.3389/fmed.2021.797171

**Published:** 2021-11-24

**Authors:** Takayuki Niitsu, Tomoki Kuge, Kiyoharu Fukushima, Yuki Matsumoto, Yuko Abe, Masashi Okamoto, Kako Haduki, Haruko Saito, Tadayoshi Nitta, Akira Kawano, Takanori Matsuki, Daisuke Motooka, Kazuyuki Tsujino, Keisuke Miki, Shota Nakamura, Hiroshi Kida, Atsushi Kumanogoh

**Affiliations:** ^1^Department of Respiratory Medicine and Clinical Immunology, Osaka University Graduate School of Medicine, Osaka, Japan; ^2^Department of Respiratory Medicine, National Hospital Organization Osaka Toneyama Medical Center, Osaka, Japan; ^3^Laboratory of Host Defense, World Premier Institute Immunology Frontier Research Center (WPI-IFReC), Osaka University, Osaka, Japan; ^4^Department of Infection Metagenomics, Genome Information Research Center, Research Institute for Microbial Diseases, Osaka University, Osaka, Japan

**Keywords:** pleural effusion, rapidly growing mycobacteria, nontuberculous mycobacteria, *Mycolicibacterium mageritense*, immunocompetent

## Abstract

*Mycolicibacterium mageritense (M. mageritense)* is a rare species among rapidly growing mycobacteria, and *M. mageritense* pleurisy is very rare. Here, we report for the first time, an immunocompetent patient with pleurisy caused by *M. mageritense*. The patient had no history of immunodeficiency and no recurrence of lung cancer after surgery. However, 8 months after surgery, he developed a new lung shadow and pleurisy. Although whole-genome analysis of the colony cultured from the patient's pleural fluid revealed *M. mageritense*, we could not identify it in time, resulting in a poor outcome. *M. mageritense* pleurisy in this case might have occurred via a bulla rupture of the lung lesion because computed tomography of the patient's chest showed pneumothorax and a lung lesion in contact with thoracic cavity. This case emphasized that nontuberculous mycobacterial pleurisy should be considered in the differential diagnoses of pleural effusion even in immunocompetent patients. Advancement of comprehensive and rapid analyses of genomic data from clinical specimens will lead to better treatment strategies.

## Introduction

Nontuberculous mycobacteria (NTM) consist of over 200 species and subspecies. They can cause infectious diseases in humans of all ages at both pulmonary and extrapulmonary sites. In Asia, 31% of NTM-associated infectious diseases are caused by rapidly growing mycobacteria (RGM) (1, 2) and usually require long-term treatment with multidrug antibiotic regimens. They are often refractory to treatment and have a high likelihood of relapse (3).

*Mycolicibacterium mageritense* (*M. mageritense*) is a rare RGM related to *Mycobacterium fortuitum* (*M. fortuitum)*, a low-virulence species that is the most common species among RGMs (4). *M. mageritense* has never been recognized as a highly pathogenic bacterium that causes serious infectious diseases. Several studies have reported cases of skin and soft tissue infections, pneumonia, and health care-associated infections among patients with or without immunodeficiency (5–7).

NTM pleurisy is very rare, and only one case of pleurisy due to *M. mageritense* has been reported (8). Here, we report for the first time, a case of severe pleurisy due to *M. mageritense* in an immunocompetent patient.

## Case Description

**Figure 1:**
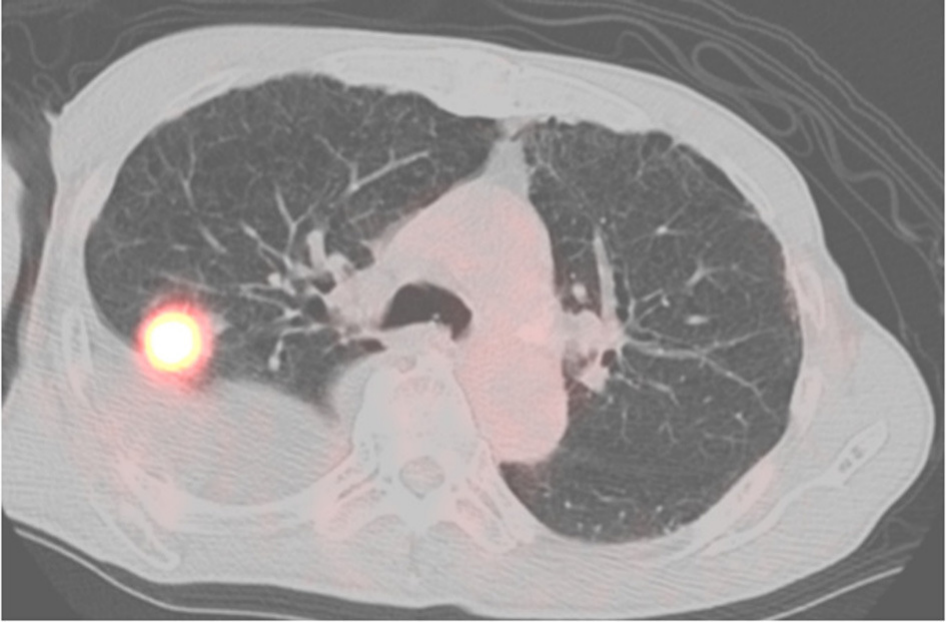
PET-CT scan showing no FDG uptake in the pleura.

A 77-year-old man was referred to our hospital for right pleural effusion lasting several months with unknown etiology. During follow-up at our hospital, we observed a nodule in the upper lobe of the right lung, showing high 2-deoxy-2-[18F] fluoro-D-glucose (FDG) uptake [Primary tumor standardized uptake value (SUV)_max_ = 12.4] on positron emission tomography-computed tomography (PET-CT) (Figure 1). After excluding malignant pleural effusion via cytological testing, we diagnosed the patient with stage IA3 squamous cell lung carcinoma and performed combined resection of the right upper lobe and part of the middle and lower lobes via video-assisted thoracic surgery. The patient was a heavy smoker (60 packs/year), with a history of radiation-treated laryngeal cancer. He had complications of chronic obstructive pulmonary disease and angina, which were treated with coronary artery stenting.

Pleural fluid analysis at surgery showed the following: total protein, 4.8 g/dL; glucose, 85.6 mg/dL; carcinoembryonic antigen, 2.6 ng/mL; neutrophils, 2.0%; lymphocytes, 78.0%; monocytes, 12.0%; eosinophils, 0.0%; adenosine deaminase, 21.0 U/L; and lactate dehydrogenase (LDH), 140 U/L, with negative bacterial and acid-fast bacterial cultures.

**Figure 2:**
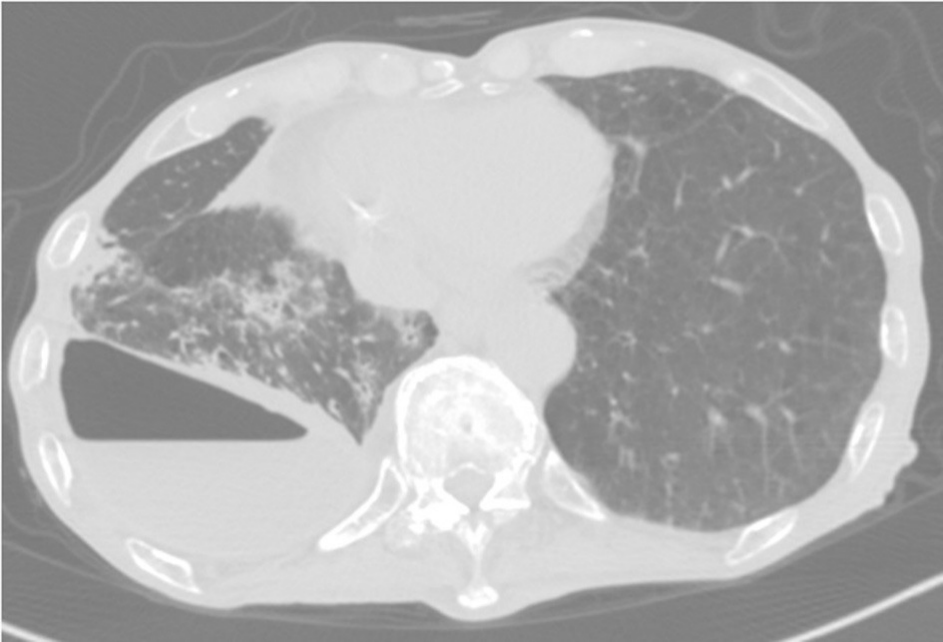
CT scan showing pneumothorax and centrilobular nodular shadows with ipsilateral increasing pleural effusion.

Eight months postsurgery, he lost his appetite. A CT scan showed increased pleural effusion with pneumothorax and new centrilobular nodular shadows in contact with the pleura (Figure 2). This time, the acid-fast bacterial smear test was positive. We diagnosed him with NTM pleurisy after excluding tuberculosis via PCR. However, we could not correctly identify the species using conventional methods such as AccuProbe (Gen-Probe Inc., San Diego, CA, USA), COBAS AMPLICOR (Roche Diagnostic, Tokyo, Japan), and a DNA-DNA hybridization assay (Kyokuto Pharmaceutical Industrial, Tokyo, Japan), although the growth and appearance of the colonies suggested *M. fortuitum*, a low-virulence organism. Table 1 shows the susceptibility results for this strain. We tried treating the pleurisy by drainage and single antibiotics, levofloxacin, and imipenem/cilastatin at the time of exacerbation. However, we could not control the effusion, and the patient died of aspiration pneumonia and CO_2_ narcosis.

**Table 1:** Susceptibility of the isolate.

**Antibiotics**	**MIC (μg/mL)**	**Susceptibility**
Clarithromycin	16	R
Azithromycin	>32	R
Cefoxitin	16	S
Imipenem	<0.5	S
Meropenem	2	S
Faropenem	4	
Amikacin	4	S
Tobramycin	>16	R
Minomycin	2	I
Doxycycline	4	I
Linezolid	<4	S
Moxifloxacin	<0.25	S
Ciprofloxacin	<0.5	S
Levofloxacin	<0.5	S
Sulfamethoxazole–Trimethoprim	<2	S

*MIC, minimal inhibitory concentration; S, susceptible; I, intermediate; R, resistant*.

Later, whole-genome sequencing revealed that his pleural effusion culture isolate was M. mageritense. The sequencing was performed following the method described before using the NovaSeq 6000 platform (Illumina, San Diego, CA, USA) (9). We used mlstverse software (9) for accessory-genome multilocus sequence type analysis and found only one profile matching *M. mageritense* with a score of 0.998 (Table 2).

**Table 2:** MLST score of the isolate.

**Species**	**Strain**	**Score**
*Mycolicibacterium mageritense*	CIP 104973	0.998

## Discussion

*M. mageritense* was originally discovered from human sputum in 1997 (10) and has been detected from human samples collected from surgical wounds, blood, sinuses, and joint fluid (11). As with other NTM species, *M. mageritense* rarely develops into pleurisy, and only one case of pleurisy caused by *M. mageritense*, which occurred in an immunocompromised host, has been reported (8). Our case is the first to have occurred in an immunocompetent host.

In the NTM pleurisy pathogenesis, two possible mechanisms are considered. One is the direct extension of lung lesions into the pleura. The other is a hematogenous route (12). Because our patient's chest CT showed pneumothorax and centrilobular shadows contacting the thoracic cavity, which were previously unseen, *M. mageritense* pleurisy in our patient might have occurred via bulla rupture of a lung lesion. We also investigated the possibility that thoracic surgery might have caused the infection. However, his pleural effusion did not increase over several months postsurgery; therefore, we think this possibility was very low. Although we could not rule out disseminated NTM, the patient had no abnormal findings other than lung disease.

In treating rare mycobacterial diseases such as those caused by *M. mageritense*, we usually determine treatment regimens by referring to previous case reports about the organism or established treatment regimens for related organisms, then modify the regimen individually as per drug-susceptibility tests. Precisely identifying the pathogen is the first step. However, due to the lack of a clinically available identification technique, we could not identify *M. mageritense* in time in this case. Advancement of comprehensive and rapid analysis of genomic data from clinical specimens will lead to clinical sequencing in NTM and thus will help clinicians evaluate the pathogenicity and choose the proper treatment timing and regimen.

In conclusion, this report describes an immunocompetent patient with both early-stage lung cancer and pleural effusion caused by *M. mageritense*. In patients with pleural effusion, RGM pleurisy should be considered as a differential diagnosis, even in patients who are not immunocompromised. Accurately identifying rare organisms using genomic data may enable establishing proper treatment strategies.

## Data Availability Statement

The datasets supporting the conclusions of this article are included within the article. Whole genome sequence analysis was deposited to BioProject (PRJDB12517), BioSample (SAMD00414014), Nucleotide (BPWM01000001-BPWM01000192).

## Author Contributions

TNii and TK drafted the manuscript. KF supervised the writing of the manuscript and was responsible for the clinical data. YA and MO contributed to critically reviewing the manuscript. HK organized and contributed to managing the case report. KH, HS, TNit, and AKa performed the mycobacterial culturing and analyzed the culture isolates. YM, DM, and SN performed the whole-genome analysis. All authors contributed to writing the final manuscript.

## Funding

This work was supported in part by AMED (grant numbers JP20fk0108129, JP21fk0108129h0702, JP21lm0203007), a GSK Research grant (grant number A-32), JSPS KAKENHI (grant numbers JP21K16118, JP21K08194), the Smoking Research Foundation, Takeda Science Foundation, and the Japan Intractable Diseases (Nanbyo) Research Foundation (grant number 2020B02).

## Conflict of Interest

The authors declare that the research was conducted in the absence of any commercial or financial relationships that could be construed as a potential conflict of interest.

## Publisher's Note

All claims expressed in this article are solely those of the authors and do not necessarily represent those of their affiliated organizations, or those of the publisher, the editors and the reviewers. Any product that may be evaluated in this article, or claim that may be made by its manufacturer, is not guaranteed or endorsed by the publisher.
